# Biochemical Characterization of the Copper Nitrite Reductase from *Neisseria gonorrhoeae*

**DOI:** 10.3390/biom13081215

**Published:** 2023-08-04

**Authors:** Daniela S. Barreiro, Ricardo N. S. Oliveira, Sofia R. Pauleta

**Affiliations:** 1 Microbial Stress Lab, UCIBIO—Applied Molecular Biosciences Unit, Department of Chemistry, NOVA School of Science and Technology, Universidade NOVA de Lisboa, 2829-516 Caparica, Portugal; 2 Associate Laboratory i4HB—Institute for Health and Bioeconomy, NOVA School of Science and Technology, Universidade NOVA de Lisboa, 2829-516 Caparica, Portugal

**Keywords:** copper nitrite reductase, AniA, *Neisseria*, copper enzyme, nitrite reduction, T1 copper center, T2 copper center, thermostability, spectroscopy, hyperthermostable

## Abstract

The copper-containing nitrite reductase from *Neisseria gonorrhoeae* has been shown to play a critical role in the infection mechanism of this microorganism by producing NO and abolishing epithelial exfoliation. This enzyme is a trimer with a type 1 copper center per subunit and a type 2 copper center in the subunits interface, with the latter being the catalytic site. The two centers were characterized for the first time by EPR and CD spectroscopy, showing that the type 1 copper center has a high rhombicity due to its lower symmetry and more tetragonal structure, while the type 2 copper center has the usual properties, but with a smaller hyperfine coupling constant (A_//_ = 10.5 mT). The thermostability of the enzyme was analyzed by differential scanning calorimetry, which shows a single endothermic transition in the thermogram, with a maximum at 94 °C, while the CD spectra in the visible region indicate the presence of the type 1 copper center up to 80 °C. The reoxidation of the *N. gonorrhoeae* copper-containing nitrite reductase in the presence of nitrite were analyzed by visible spectroscopy and showed a pH dependence, being higher at pH 5.5–6.0. The high thermostability of this enzyme may be important to maintaining a high activity in the extracellular space and to making it less susceptible to denaturation and proteolysis, contributing to the proliferation of *N. gonorrhoeae*.

## 1. Introduction

Nitrite reductases (NiRs) are copper- or heme-containing enzymes that catalyze the reduction of nitrite (NO_2_^−^) to nitric oxide (NO) in the periplasm of gram-negative bacteria [1], according to Equation (1), as the second step of the denitrification pathway:

NO_2_^−^ + e^−^ + 2 H^+^ → NO + H_2_O (1)

Heme-containing enzymes, *cd*_1_NiRs, are homodimers, with each subunit containing one *d*_1_-type heme and one *c*-type heme, with the first being the catalytic site and the second being responsible for accepting electrons from small redox proteins, such as *c*-type cytochromes or cupredoxins, and channeling them to the active site [2,3,4]. Copper-containing enzymes, CuNiRs, have two copper centers with functions similar to these two hemes. Electrons enter through the type 1 copper center (T1Cu) and are then transferred to the type 2 copper center (T2Cu), where NO_2_^−^ is converted to NO [5,6,7,8,9].

**Figure 1:**
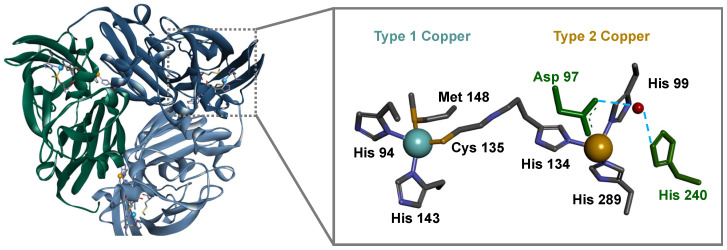
Structure of CuNiR from *Neisseria gonorrhoeae* as a trimer, evidencing the location of T1Cu center (blue sphere) in each subunit and T2Cu center (brown sphere) in the interface between the subunits. The coordination of the two centers is shown in the right-hand panel. Carbon, nitrogen, and sulfur are colored in dark grey, blue, and yellow, respectively. The aspartate and histidine residues involved in the catalytic cycle are colored green. Figures were prepared in Discovery Studio Visualizer using the coordinates PDB ID 1KBW. Numbering of the residues is according to the mature sequence of *N. gonorrhoeae* CuNiR.

In recent years, with the advances in genome sequencing, the gene encoding CuNiRs has been shown to be associated with additional domains at either the N- or the C-terminus. These additional domains bind either *c*-type hemes or T1Cu centers and mediate electron transfer to the core domains [10]. This core domain has a homotrimeric structure, with each subunit containing the two copper centers, one T1Cu and one T2Cu (Figure 1), with different structural, spectroscopic, and functional properties [11]. The T1Cu copper is coordinated by two histidine residues, one methionine, and one cysteine, from the same subunit. T2Cu, the active site, is located at the interface between the subunits and it is coordinated by three histidine side chains, one of which is from the other subunit (Figure 1) [12]. These two centers are connected by a conserved cysteine–histidine bridge consisting of the coordinating cysteine of the T1Cu and the histidine of the T2Cu (Figure S1 in the Supplementary Materials). Two other conserved residues in the catalytic pocket have been reported to play an important role in catalysis, Asp97 and His240 (numbering according to *Neisseria gonorrhoeae* CuNiR) [13,14,15], which are H-bonded to a bridging water molecule (Figure 1). These conserved residues are involved in the formation of the T2Cu^+^-NO_2_^−^ intermediate species, by triggering the electron transfer through the cysteine–histidine bridge, thus acting as a “substrate-sensing pathway” [7,8,16,17,18].

CuNiRs are divided into three classes, blue, green, and bluish-green, according to the spectroscopic properties of their T1Cu center [11], since the T2Cu center does not significantly contribute to the absorption spectrum in the visible region. The absorption spectrum of the CuNiRs shares a band with a maximum around 600 nm, corresponding to a S(Cys) 3pπ → Cu 3dx^2^–y^2^ charge-transfer transition [19]. CuNiRs with these singular spectral features belong to the blue CuNiR class. However, most of the isolated NiRs belong to the bluish-green class and have an additional absorption band with a maximum around 450 nm, which is attributed to a S(Cys) 3p pseudo σ → Cu 3dx^2^–y^2^ charge-transfer transition [19]. In the absorption spectrum of the green CuNiRs, in addition to the absorption band at 600 nm having a lower intensity, compared to the absorption band centered at 450 nm, this absorption band has a shoulder at 400 nm that has been assigned to an S(Met) → Cu charge-transfer transition. In the case of cupredoxins, a more intense absorption at 450 nm is correlated with a shorter Cu—S(Met) bond (and with a longer Cu—S(Cys) bond) [19,20], but in CuNiRs this is not the case, as the Cu—S(Met) bond is relatively short for all classes (≤2.6 Å) [21]. 

*N. gonorrhoeae* is an obligate human pathogen responsible for the sexually transmitted disease gonorrhea, which affects millions of people worldwide. To date, there is no effective vaccine against *N. gonorrhoeae* available, and strains resistant to the antibiotics used to treat it are on the rise, making the study of its survival mechanisms important as they represent good drug targets [22,23,24]. One of these mechanisms is the anaerobic respiration. *N. gonorrhoeae* possesses a truncated denitrification pathway, consisting of a CuNiR (here referred to as *Ng*CuNiR, and also known as AniA in the literature) and a nitric oxide reductase, qNOR, which allows the use of N-based compounds as alternative electron acceptors for energy conservation [25]. In addition, considering that NO is a modulating molecule of the immune response, as low levels (nM) of NO are anti-inflammatory and high levels of NO (μM) are pro-inflammatory [26], qNOR will be able to lower these levels [27]. Moreover, *Ng*CuNiR has been shown to play an important role in infection by producing NO and initiating a eukaryotic signaling pathway that leads to the suppression of epithelial exfoliation [28,29].

*Ng*CuNiR is a copper nitrite reductase that belongs to the bluish-green class according to its visible spectra [15]. The proposed electron transfer pathway to *Ng*CuNiR starts at the inner membrane respiratory chain from the *bc*_1_ complex to the terminal cytochrome *cbb*_3_, where the electrons are deviated from the *cbb*_3_ O_2_-reduction subunits to *Ng*CuNiR via a physiological electron donor in the periplasm [30]. In addition, a periplasmic copper chaperone, AccA, is reported to be responsible for the metallation of *Ng*CuNiR [31]. Thus, *Ng*CuNiR should be located in the periplasm of *N. gonorrhoeae* to be metallated and to receive electrons. In fact, *Ng*CuNiR is described as a periplasmic enzyme, anchored to the outer membrane by a palmitate bound to a cysteine residue [32]. However, whole-cell immunoblotting and immune-scanning electron microscopy have shown that it is also exposed at the surface [33,34]. The cell surface location of *Ng*CuNiR explains the presence of antibodies in the sera of patients with gonococcal infections [35].

The redox partner of this enzyme is still unknown, and it has been shown not to be the lipid-modified azurin from the same organism [36], though this protein is also bound to the outer membrane and it is the redox partner of another outer membrane-attached enzyme, the bacterial peroxidase [37,38].

Here, we report the first stability study of *Ng*CuNiR using differential scanning calorimetry, circular dichroism, and visible spectroscopy, which indicates that this is a highly stable protein. In addition, the EPR and CD spectral features are described for the first time and correlated with its visible spectrum and the geometry of the T1Cu center. Given the importance of this enzyme during infection, it represents an excellent target for the design of novel therapies, and thus the knowledge of its stability and spectroscopic properties can help identify compounds that either destabilize its structure or interact with the active site.

## 2. Results and Discussion

### 2.1. Protein Production

The soluble domain of *Ng*CuNiR was heterologously produced in *E. coli* as a cytoplasmic 36.5 kDa protein, since a signal peptide is missing in the construct used (Figure S1 in the Supplementary Materials). The enzyme was purified in two chromatographic steps: an affinity chromatography (since it has a His-tag at the C-terminus, Figure S2 in the Supplementary Materials) followed by an anionic chromatography (since the pI of *Ng*CuNiR is 5.7, considering the primary sequence of the construct).

**Figure 2:**
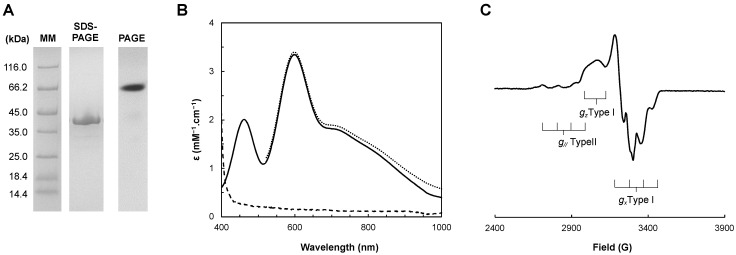
(**A**) Electrophoretic profile of the purified *Ng*CuNiR in a 12.5% SDS-PAGE and 10% PAGE stained with Coomassie blue. Legend: MM—Molecular Marker. (**B**) Visible spectra of *Ng*CuNiR in 10 mM Tris-HCl, pH 7.6. Legend: dashed, solid, and dotted lines represent the ferricyanide-oxidized, as-isolated, and dithionite-reduced spectra, respectively. (**C**) X-band EPR spectrum of 400 μM *Ng*CuNiR in 10 mM Tris-HCl, pH 7.6 acquired at 30 K.

The protein was judged pure by its SDS-PAGE and PAGE (Figure 2A) and had a purity ratio of A_598nm_ of the oxidized state to A_277nm_ of the as-isolated state of ~0.1. This purification procedure yielded 15–30 mg pure *Ng*CuNiR/L of culture medium. The copper content per monomer was determined to be 2.0 ± 0.1, confirming that *Ng*CuNiR was produced with both copper centers occupied.

The PAGE of the purified fraction shows a single band, implying that the sample is homogeneous and there is no mismetallation. The apparent molecular weight of *Ng*CuNiR is 96 ± 10 kDa (Figure S3 in the Supplementary Materials), indicating that it behaves as a homotrimer in solution, as expected according to its X-ray structure (Figure 1) [15].

### 2.2. Spectroscopic Characterization

#### 2.2.1. UV–Visible Spectroscopy

The visible spectrum of *Ng*CuNiR is presented in Figure 2B. The as-isolated *Ng*CuNiR has the typical features of CuNiRs with two absorption bands with maxima at 460 nm and 598 nm, and a A_460nm_/A_598nm_ of 0.6 as previously reported [15]. In the oxidized spectrum, by the addition of an excess of sodium ferricyanide, the band at 598 nm slightly increases in intensity (the band at 460 nm is masked by the ferricyanide absorption), whereas in the presence of sodium dithionite the absorption bands completely disappear.

Copper quantification was used to determine the molar extinction coefficient (per monomer) of 2.06 ± 0.02 mM^−1^·cm^−1^ and 3.44 ± 0.02 mM^−1^·cm^−1^ at 460 nm and 598 nm, respectively, at pH 7.6 (Figure 2B). These values and their ratio are closer to the ones reported for the bluish-green CuNiR from *Bradyrhizobium japonicum*: 2.6 mM^−1^·cm^−1^ (458 nm) and 4.4 mM^−1^·cm^−1^ (592 nm), which has a ε_458nm_/ε_592nm_ of 0.59 [39], as well as to other bluish-green CuNiRs [40,41].

These spectral features are dictated by the geometry of the T1Cu center of *Ng*CuNiR. The absorption band with a maximum at 598 nm is assigned to a S(Cys) 3pπ → Cu charge-transfer transition, while the one with a maximum at 460 nm is assigned to a S(Cys) pseudo σ → Cu charge-transfer transition [11,19,20,42]. The relative intensity of these two absorption bands determines the class of the CuNiR, as blue CuNiRs have a very weak absorption band at 450 nm and green CuNiRs have an absorption band at 450 nm that is stronger than the absorption band at 600 nm, with a ratio of ε_450nm_/ε_600nm_ > 1 (as in the case of *Achromobacter cycloclastes* CuNiR with A_464nm_/A_590nm_ of 1.3 [43]), while bluish-green CuNiRs have a relatively intense 450 nm band but a ε_450nm_/ε_600nm_ < 1 [10,21]. In the visible spectrum, there is also a broad band centered at around 750 nm, assigned to d → d transitions, while the band around 400 nm, assigned to the S(Met) → Cu charge-transfer transition [44], and present in green CuNiRs, is absent (*vide infra*). This confirms that *Ng*CuNiR is a bluish-green CuNiR.

#### 2.2.2. Electron Paramagnetic Resonance Spectroscopy

The 30 K electron paramagnetic resonance (EPR) spectrum of *Ng*CuNiR has features of the presence of T1Cu and T2Cu centers (Figure 2C). The T2Cu center has the typical features of an axial signal (*g*_⊥_ = 2.065 and *g*_//_ = 2.346 with A_//_ = 10.5 mT) like that of *Pseudomonas chlororaphis* CuNiR (*g_x,y,z_* = 2.040, 2.110, 2.350 with A_z_ = 10.7 mT) [45] and *Rhodobacter sphaeroides* CuNiR (*g*_//_ = 2.34; A_//_ = 12.5 mT) [40]. The T1Cu center has a rhombic signal, with a small hyperfine coupling constant giving rise to a partially resolved hyperfine structure in the *g*_z_ region. The *Ng*CuNiR T1Cu signal has *g*_z_ = 2.20 (A_z_ = 2.0 mT), *g*_y_= 2.055 (A_y_ = 5.0 mT), and *g*_x_ = 2.023 (A_x_ = 7.2 mT), which are similar to the *g*-values of pseudoazurin [42], stellacyanin [46], and cucumber basic protein [47].

The *Ng*CuNiR EPR spectrum differs from that of blue and green CuNiRs [6,43,48,49], as well as from other bluish-green CuNiRs [39,40,41], and is unique among these enzymes. In cupredoxins, an increase in the intensity of the absorption band with a maximum at 450 nm, arising from the S(Cys)pseudo σ → Cu charge-transfer transition, corresponds to a more rhombic EPR signal [11,19,42]. However, this is not observed in CuNiRs. In fact, the bluish-green CuNiR from *B. japonicum*, with a similar visible spectrum to *Ng*CuNiR, shows a different EPR spectrum, with nearly axial signals attributed to both T1Cu and T2Cu [39]. Nevertheless, the *g*-values for the *g*_z_ of both signals are similar to those observed in *Ng*CuNiR. Thus, *Ng*CuNiR combines the typical rhombicity of the EPR signals of the T1Cu centers of bluish-green cupredoxins (such as pseudoazurin, stellacyanin, and cucumber basic protein) together with an EPR of the T2Cu center with a smaller hyperfine constant than those of other CuNiRs (*vide infra*).

#### 2.2.3. Circular Dichroism

**Figure 3:**
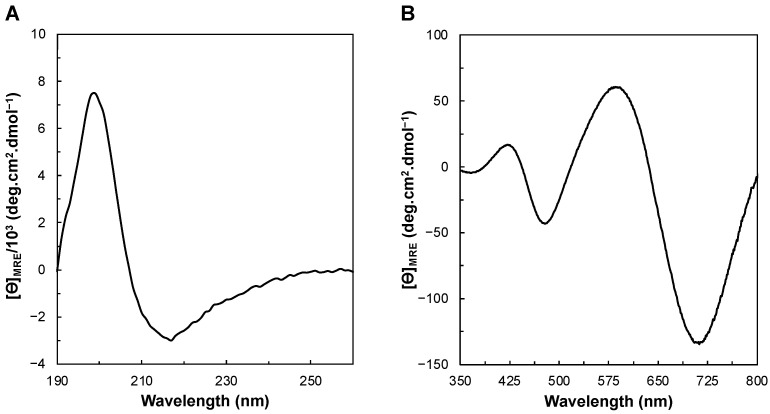
Circular dichroism spectra of *Ng*CuNiR in the far-UV (**A**) and visible (**B**) regions, at 20 °C.

Circular dichroism (CD) in the far-UV region (190–260 nm) provides information about the folding status and secondary structure content of a protein. The CD spectrum of the isolated *Ng*CuNiR in the far-UV region has typical features of a folded protein composed mainly of β-sheets, since it has a positive peak at 200 nm and a negative peak at 217 nm (Figure 3A) [50]. This spectrum is similar to the ones of the green fluorescence protein [51] and the intestinal fatty acid binding protein [52,53] that are known to be composed mainly of β-sheets.

The analysis of the far-UV spectrum at 20 °C using either the BeStSel [54,55] or the DichroWeb [56,57] algorithms estimated that *Ng*CuNiR is composed mainly of β-sheets (37.9% β-sheets and 3.2% α-helices, or 41.3% β-sheets and 5.3% α-helices, using BeStSel or DichroWeb, respectively). These values agree with the analysis of coordinates of this protein (39.1% β-sheets and 8.3% α-helices, PDB ID 1KBW, Figure 1), and it is also consistent with the low ellipticity of the spectrum [50].

The CD spectrum in the visible region arises from prosthetic groups. Thus, the coordination and geometry of the T1Cu center and its neighboring residues will influence the observed bands [58]. The CD spectrum in the visible region has two positive peaks at 422 nm and 588 nm, and two negative peaks at 478 nm and 712 nm. Another maximum with a positive ellipticity seems to form around 800 nm, although data were not collected at higher wavelengths to confirm it. According to other studies reported on CuNiRs and blue copper proteins, these peaks arise mainly from charge-transfer transitions (S(Cys) → Cu) and d → d transitions of the T1Cu center. The charge-transfer transitions S(Cys) pseudo σ → Cu and S(Cys) π → Cu have been reported from 457 nm to 538 nm and 571 nm to 593 nm, respectively, whereas the d → d transitions are observed between 671 nm and 893 nm [44,47,59,60]. Therefore, the maxima ellipticity at 478 nm and 588 nm are assigned to S(Cys) pseudo σ → Cu and S(Cys) 3pπ → Cu, respectively, and the maximum at 712 nm to d → d transitions [44]. Another main transition, reported in blue copper proteins and in green CuNiRs between 390 and 427 nm, is attributed to an S(Met) → Cu charge-transfer transition [44]. The experimental data do not provide a clear hint that the peak observed at 422 nm corresponds to this transition since the absorption band with a maximum at 460 nm in the absorption spectrum of *Ng*CuNiR is quite symmetric (Figure 2B). However, one cannot exclude that there are two bands centered at 422 nm and 478 nm contributing to the absorption band observed at 460 nm.

The *Ng*CuNiR’s CD spectrum in the visible region is dominated by the S(Cys) 3pπ → Cu transition with a positive ellipticity and by the S(Cys) pseudo σ → Cu transition with a negative ellipticity, like that of the green *R. sphaperoides* CuNiR [44]. However, it differs from the spectrum of this protein, as the most intense transition is the one centered at 588 nm, a S(Cys) 3pπ → Cu charge-transfer transition.

The T2Cu center is reported to make very small contributions to the visible region of the CD spectrum of CuNiRs, as the spectrum of variant proteins depleted of this center showed insignificant changes when compared to the native one [44,60]. Spectral features of the T2Cu center have been observed above 900 nm, with bands around 1000 nm being assigned to ligand field transitions.

The T1Cu center of *Ng*CuNiR is coordinated by two histidines, one cysteine and one methionine, and its spectral features arise from charge-transfer transitions between the copper atom and the sulfur atom of its coordinating cysteine. These transitions are directly influenced by the bond length between these atoms, which depends on the geometry of the center [19,20,42]. The T1Cu center of *Ng*CuNiR has a shorter Cu—S(Met) bond (2.61 Å) when compared to cupredoxins (such as pseudoazurin from *Paracoccus pantotrophus*, [61], Table S1 in the Supplementary Materials), but this bond is slightly longer than that observed in other CuNiRs (Table S1 in the Supplementary Materials and [21]). However, its Cu—S(Cys) is the shortest among the CuNiRs (1.90 Å) (Table S1 in the Supplementary Materials and [21]), which may explain some of its unique spectroscopic properties.

In addition, the superposition of the T1Cu centers of the three classes of CuNiR highlights other differences (Figure S4 in the Supplementary Materials). The His_2_-Cu-Met and Cys-Cu-Met angles are 128° and 108°, respectively, as for the green CuNiR from *A. cycloclastes*, but the side-chain conformation of the Met148, which coordinates the T1Cu in *Ng*CuNiR, adopts a “*gauche*” conformation. Such a conformation is also observed in other bluish-green CuNiRs [21] and in blue CuNiRs (Figure S4 in the Supplementary Materials), and differs from the “*trans* (*anti*)” conformation of the Met ligand in green CuNiRs [21]. Apart from the side-chain conformation of the Met ligand, the positions of the coordinating atoms of the T1Cu center of green CuNiRs and *Ng*CuNiR are very similar, with the exception of CysS^γ^ (Figure S4 in the Supplementary Materials). Comparison of the dihedral angles between the His_1_-Cu-His_2_ and Cys-Cu-Met planes of CuNiRs (Table S1 in the Supplementary Materials) shows that the T1Cu center of *Ng*CuNiR has the smaller angle, indicating a more distorted center. Since discrepancies between these values have been reported between damage-prone and damage-free structures [62], these differences and their contribution to the electronic properties of the T1Cu center need to be re-analyzed, as suggested by C. Brondino in [63].

In conclusion, the spectral features of *Ng*CuNiR support the observation that the T1Cu center has a lower symmetry and a more tetragonal structure, with a shorter Cu—S(Cys) bond and an extended Cu—S(Met) bond, which is still shorter than that observed in cupredoxins. These features lead to the unique spectroscopic feature of having a T1Cu center with a rhombic EPR signal.

These electronic properties of the copper centers of *Ng*CuNiR may be functionally relevant by tuning the reduction potential of the T1Cu center and the electron transfer rate to the catalytic T2Cu center. These aspects will be pursued in future studies of the enzyme.

### 2.3. Thermostability

**Figure 4:**
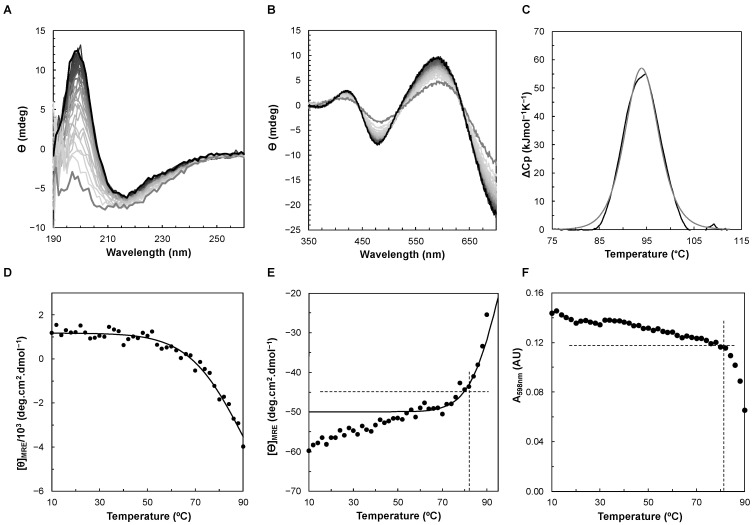
Thermal stability of *Ng*CuNiR monitored by CD, visible spectroscopy, and DSC. Temperature ramp (from 10 °C to 90 °C) of *Ng*CuNiR in the far-UV region (**A**) and visible region (**B**). (**C**) Thermogram of *Ng*CuNiR obtained by DSC (black line), fitted to a one-peak transition model (grey line). Mean residue ellipticity ([ϴ]_MRE_) at 205 nm (**D**) and 480 nm (**E**) as a function of temperature. The experimental data were fitted using the equations for a two-state transition model (solid black line). (**F**) Variation of the absorbance at 598 nm as a function of temperature. The dashed lines in (**E**,**F**) mark the point at which the ellipticity and the absorbance, respectively, begin to change abruptly.

The thermostability of *Ng*CuNiR was assessed by CD in the visible and far-UV regions, absorption spectroscopy, and differential scanning calorimetry (DSC) (Figure 4), which provide complementary information. The DSC gives information on the overall unfolding process, while the CD in the far-UV region monitors changes in the secondary structure content of the protein. The CD acquired in the visible region and absorption spectroscopy will monitor changes in the metal center, in this case the T1Cu center, which is not covalently bound to the polypeptide chain.

The thermogram obtained by DSC shows a single sharp endothermic peak that was fitted to a one-peak transition model, from which a transition temperature, T_M_, of 94.0 ± 0.1 °C, and a van’t Hoff enthalpy of unfolding, ΔH_vH_, of 542 ± 2 kJ/mol, were estimated (Figure 4C). Since the ΔH_cal_/ΔH_vH_ ratio was 0.96 (ΔH_cal_ was 519 ± 2 kJ/mol), it is plausible to assume that the two-state unfolding model is valid. Nevertheless, it cannot be excluded that the unfolding process may involve an intermediate state, as shown for *Alcaligenes faecalis* S-6 CuNiR [48] and suggested by the analysis of the CD in the far-UV region (*vide infra*).

This high thermostability is confirmed by the analysis of the CD in the far-UV region (Figure 4A), which shows that the secondary structure of *Ng*CuNiR remains unchanged up to 50 °C (Figure 4A,D). At higher temperatures the spectrum loses intensity throughout the spectral window, especially at 200 nm, without the observation of an isosbestic point, suggesting the presence of an intermediate species (so that there are more than two component spectra contributing to each spectrum, although the profile of a predominantly β-sheet protein and that of a random coil state may not be superimposed) [50]. Fitting the mean residue ellipticity at 205 nm to a two-state transition model from a folded to an unfolded state, assuming an equal heat capacity for both states, estimated a T_M_ of 93 ± 2 °C and a ΔH of 88 ± 2 kJ/mol. However, this process is not reversible, as the CD spectrum at 20 °C acquired after the temperature ramp is not identical to the initial one (Figure S5 in the Supplementary Materials). Therefore, the true thermodynamic parameters of the unfolding process cannot be obtained as explained in [58], and thus the values estimated by CD cannot be compared with those obtained by DSC.

The stability of the T1Cu center was monitored by both CD and absorption spectroscopy, showing that there are two processes: a slow change in ellipticity or absorbance attributed to the S(Cys) → Cu charge-transfer transitions up to 80 °C, and then a sharp change with increasing temperature (Figure 4B,E,F and Figure S6 in the Supplementary Materials). This indicates that the stability of the T1Cu center is only slightly affected up to 80 °C, although the secondary structure starts to change more significantly above 50 °C. In fact, the CD spectra in the visible region did not disappear even at 90 °C (Figure 4), but a shift towards lower and higher energy was observed in the positive peaks at 425 nm and 588 nm, respectively. This indicates that the T1Cu coordination sphere is maintained to some extent up to this temperature. This agrees with the EPR spectra obtained at different temperatures for *A. faecalis* S-6 CuNiR [48], which show features of the presence of this center up to 90 °C. The shifts observed in the peaks can be explained by the change in the geometry of the center, with changes in the bond distances between the Cu atom and the S ligands (Met and Cys). The mean residue ellipticity at 480 nm, from 50 to 90 °C, was fitted with a T_M_ of 96 ± 1 °C and a ΔH of 161 ± 3 kJ/mol (Figure 4E).

The T_M_ and ΔH of *Ng*CuNiR is close to that of *A. faecalis* S-6 CuNiR, which has a T_M_ of 99.6 °C and a ΔH of 1630 kJ/mol [48]. This high thermostability seems to be a feature of these enzymes, which may be due to their β-sheet structural motif. Moreover, the enthalpy of denaturation determined by CD is lower than that estimated by DSC, although this is attributed to the fact that in the CD the model assumes that there is no difference in the heat capacity between the folded and the unfolded states of *Ng*CuNiR, leading to an underestimation of this thermodynamic parameter [50].

### 2.4. Reoxidation of NgCuNiR in the Presence of Nitrite

**Figure 5:**
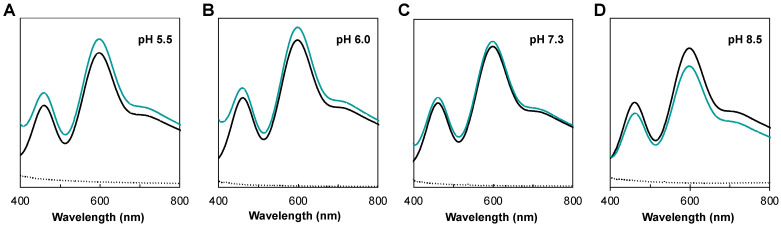
Reoxidation of dithionite-reduced *Ng*CuNiR upon addition of sodium nitrite at (**A**) pH 5.5, (**B**) 6.0, (**C**) 7.3, and (**D**) 8.5. Visible spectra of as-isolated *Ng*CuNiR are represented as black lines, dithionite-reduced as dotted black lines, and reoxidized after 30 min incubation with nitrite as blue lines. The as-isolated *Ng*CuNiR is 10% reduced.

The reoxidation of *Ng*CuNiR in the presence of nitrite was evaluated by visible spectroscopy at different pH values (5.5, 6.0, 7.3 and 8.5) (Figure 5). In these studies, the reoxidation of the T1Cu center upon addition of nitrite in the presence of an excess of reducing agent was monitored by analyzing the increase in intensity of the absorption bands at 460 nm and 598 nm.

The addition of reducing agent resulted in the complete disappearance of the T1Cu absorption bands of *Ng*CuNiR, confirming the reduction of this center (Figure 5, black line). Upon the addition of nitrite, both bands reappeared, indicating T1Cu reoxidation due to electron transfer to the T2Cu for nitrite reduction (Figure 5, blue line). The reoxidation of the dithionite-reduced *Ng*CuNiR is higher at pH 5.5–6.0, with the complete reoxidation of the T1Cu after 30 min (Figure 5A,B). For assays performed at the other pH values, there is a decrease in the amount of reoxidized *Ng*CuNiR, especially at pH 8.5 (Figure 5D). At this pH, there is 78% reoxidation of *Ng*CuNiR. Similar results have been reported for *B. japonicum* CuNiR [39]. In fact, higher activity is expected at acidic pH because nitrite reduction requires protons (Equation (1)).

## 3. Materials and Methods

### 3.1. Chemicals and Solutions

All chemicals were pro-analysis grade purchased from Merck (Merck, Lowe, NJ, USA), Sigma-Aldrich (Merck, Lowe, NJ, USA), PanReac AppliChem GmbH (Darmstadt, Germany), Honeywell Riedel-de-Haën and Fluka Chemie GmbH (Buchs, Switzerland) and used without further purification. All solutions for protein purification were prepared in deionized water and pre-filtered.

### 3.2. Heterologous Production of NgCuNiR

*Ng*CuNiR was heterologously produced in *Escherichia coli* BL21(DE3) competent cells (Merck, Lowe, NJ, USA). The expression vector encoding the soluble domain of *Ng*CuNiR (pET28A_*aniA*) was a kind gift from Prof. Michael E. P. Murphy (from the University of British Columbia, Canada) and the cloning protocol of *aniA* is described in [15]. Four to five colonies of *E*. *coli* BL21(DE3) cells transformed with pET28a_*aniA* were selected to inoculate 50 mL of Luria–Bertani medium, supplemented with 30 μg/mL of kanamycin sulfate (Merck, Lowe, NJ, USA) and grown overnight at 37 °C, 210 rpm. Fresh 2xYT medium, supplemented with 30 μg/mL of kanamycin and 0.1 mM of CuSO_4_, was inoculated with 2% of the pre-inoculum and grown at 37 °C and 210 rpm. At an OD_600 nm_ of 0.8, IPTG was added to a final concentration of 0.5 mM and cells were grown at 25 °C and 120 rpm for 20 h. The cells were harvested at 8739× *g*, 6 °C for 15 min and resuspended in 50 mM of Tris-HCl, pH 7.6. Cells were stored at −20 °C until further use.

### 3.3. Purification of NgCuNiR

Prior to cell lysis, a cocktail of protease inhibitors (cOmplete Mini, EDTA-free Protease inhibitors, Roche, Basel, Switzerland), DNase I (Roche, Basel, Switzerland) and 5 mM CuSO_4_ were added to the cell suspension. Cells were disrupted with a French Press by subjecting the cells to a pressure of 18,000 psi. The lysed cells were centrifuged at 41,399× *g* and 6 °C for 1 h (Beckman Avanti J-26 centrifuge, Brea, CA, USA) to obtain the soluble protein extract.

The purification protocol was adapted from the one described in [15], consisting of two purification steps. The soluble extract was loaded onto an immobilized nickel-sepharose matrix (5 mL HisTrap HP, Cytiva, Marlborough, MA, USA) equilibrated with 20 mM Tris-HCl, pH 8.0 and 500 mM NaCl. *Ng*CuNiR was eluted with a gradient of imidazole between 0 to 500 mM in the equilibration buffer. The fractions containing *Ng*CuNiR were concentrated over an Amicon^®^ Ultra-15 Centrifugal Filter Unit with 30 MWCO (Millipore, Merck, Lowe, NJ, USA). The buffer was exchanged to 10 mM Tris-HCl, pH 7.6 using a Sephadex G-25 PD10 (Cytiva, Marlborough, MA, USA) desalting column, equilibrated in the same buffer. This fraction was then applied onto an anion exchange column (Resource Q, Cytiva, Marlborough, MA, USA) equilibrated with 20 mM Tris-HCl, pH 7.6. *Ng*CuNiR was eluted with an ionic strength gradient from 0 to 500 mM of NaCl in the equilibration buffer and the fractions containing *Ng*CuNiR were concentrated, and buffer exchanged to 10 mM Tris-HCl, pH 7.6 in an Amicon^®^ Ultra-15 Centrifugal Filter Unit with 30 MWCO (Millipore, Merck, Lowe, NJ, USA). The pure *Ng*CuNiR was stored at −20 °C until further use. During the purification process, the fractions collected were analyzed by UV–visible spectroscopy and in a 12.5% SDS-PAGE in Tris-Tricine buffer (electrophoresis performed at 150 V for 70 min, stained with Coomassie Blue). The protein was judged pure by its combined electrophoretic profile in the 12.5% SDS-PAGE and 10% PAGE in Tris-Tricine buffer (PAGE run at 100 V for 5 h, stained with Coomassie Blue).

Total protein was quantified using the BCA kit (Sigma, Merck, Lowe, NJ, USA), with bovine serum albumin (BSA) (Sigma, Merck, Lowe, NJ, USA) as the standard protein, and the copper content was determined using a modified version of the copper(I)-biquinoline assay [38,64].

### 3.4. Apparent Molecular Weight

The apparent molecular weight of *Ng*CuNiR was determined by size exclusion chromatography using a Superdex 200 10/300 GL column (Cytiva, Marlborough, MA, USA). The column was equilibrated with 50 mM Tris-HCl, pH 7.6 and 150 mM NaCl. Samples of *Ng*CuNiR (50 nmol) were prepared in the running buffer. The calibration curve was prepared with proteins from the Gel Filtration Calibration Kit, High Molecular Weight (Cytiva, Marlborough, MA, USA), with the protein solutions prepared in the running buffer according to the instructions.

### 3.5. Spectroscopic and Thermal Characterization

UV–visible spectra were acquired in a Shimadzu UV-1800 spectrophotometer (Shimadzu Europa GmbH, Duisburg, Germany). The molar extinction coefficients were determined based on the copper concentration. The reduced and oxidized states of *Ng*CuNiR were obtained by the addition of sodium dithionite or potassium ferricyanide, respectively.

Electron paramagnetic resonance spectra were acquired on a Bruker EMX spectrometer (Bruker Billerica, MA, USA) coupled to an Oxford Instruments ESR-900 (Oxford Cryosystems, UK) continuous flow helium cryostat and a high-sensitivity perpendicular-mode rectangular cavity. The spectra were acquired at a microwave frequency of 9.40 GHz, a microwave power of 0.202 mW, modulation amplitude of 0.5 mT and receiver gain of 1.0 × 10^5^. Other experimental conditions are listed in the figure legend.

Circular dichroism spectra were acquired in an Applied Photophysics Chirascan^TM^ qCD spectrometer (Leatherhead, Surrey, UK). The spectra in the far-UV region (190–260 nm) were acquired in a sample of 5.3 μM *Ng*CuNiR, in 20 mM sodium phosphate buffer, pH 7.0, with a 1 mm path length cuvette with a total volume of 350 μL. In the visible region (350–700 nm or 350–850 nm) the spectra were recorded for a 197 μM (temperature ramp up to 700 nm) and 502 μM (CD spectra up to 850 nm) *Ng*CuNiR sample, in 20 mM potassium phosphate buffer, pH 7.0, with a 2 mm path length cuvette with a total volume of 700 μL. The concentration of the samples was determined using the absorbance at 205 nm and the extinction coefficient of 1277 mM^−1^cm^−1^, based on the amino acid content [65], or absorbance at 598 nm and the extinction coefficient determined in this manuscript. The CD data were reported in mean residue ellipticity ([θ]_MRE_) as a function of wavelength. The [θ]_MRE_ is the CD raw data (in deg) corrected for the *Ng*CuNiR concentration of the solution using Equation (2):

(2) $${\left[\theta \right]}_{\mathrm{MRE}}=\frac{\theta \left(\deg\right)\times \mathrm{MRW}}{10\times L\times C}$$

in which MRW (mean residue weight) is the molecular mass of *Ng*CuNiR divided by the number of peptide bonds, C is the concentration of the protein in the sample in gmL^−1^, and L is the pathlength of the cell in cm.

The CD spectra between 190 and 260 nm are an average of three spectral acquisitions at 20 °C, with a bandwidth and step-size of 1 nm, and acquired with a time per point of 3 s. The far-UV data analysis to determine the secondary structure content was performed with the servers BeStSel (https://bestsel.elte.hu/, accessed on 10 June 2023) [54,55] and Dichroweb (http://dichroweb.cryst.bbk.ac.uk/, accessed on 10 June 2023) [56,57]. The CD spectra between 350 and 850 nm are an average of three spectral acquisitions at 20 °C, with a bandwidth and step-size of 1 nm, and acquired with a time per point of 2 s. The temperature-dependent CD spectra were acquired between 10 °C and 90 °C, with a stepped ramp mode of 1 or 0.5 s per point (for the far-UV or visible region, respectively), an increase of 2 °C for each measurement and a stabilization period of 1 min between each point.

The unfolding process was analyzed using the Gibbs–Helmholtz method—to fit the change of CD at a single wavelength as a function of temperature, considering a two-state transition of a folded native state to an unfolded state, and considering that the heat capacity of the folded and the unfolded states are equal, ΔCp = 0 [50,58]. For the Gibbs–Helmholtz method, the experimental data were fitted using Equations (3)–(6) and using the SOLVER Add-in program of Microsoft Excel (version 16.7). The molar ellipticity at any given temperature (T), [θ]_T_, is given by Equation (3):

(3) $${\left[\theta \right]}_{T}=\alpha \times \left({\left[\theta \right]}_{F}-{\left[\theta \right]}_{U}\right)+{\left[\theta \right]}_{U},$$

in which the fraction folded at any given temperature, α, is given by Equation (4):

(4) $$\alpha =\frac{K}{1+K}$$

and the folding constant, K, at any given temperature is given by Equation (5):

(5) $$K=\exp\left(\frac{-\Delta G}{R\times T}\right)$$

and ΔG is given by Equation (6):

(6) $$\Delta G=\Delta H\times \left(1-\frac{T}{T_{M}}\right)$$

in which T_M_ is the temperature at which the fraction folded, α, is 0.5.

Differential scanning calorimetry (DSC) was performed using a TA™ Nano DSC calorimeter (New Castle, DE, USA) and the thermogram was analyzed with NanoAnalyze™ software (version 3.12.5, TA instruments, New Castle, DE, USA) to obtain the melting temperature and thermodynamic parameters. The sample of *Ng*CuNiR was prepared at 35 µM in 20 mM potassium phosphate buffer pH 7.0 and the blank sample was the sample buffer. The thermogram was acquired between 10 and 115 °C at 6.1 bar.

### 3.6. Reoxidation of NgCuNiR in the Presence of Nitrite

Reoxidation of the reduced *Ng*CuNiR in the presence of nitrite was assessed by UV–visible spectroscopy. As-isolated *Ng*CuNiR (40–45 µM) was initially reduced with 2 mM of sodium dithionite. Then, sodium nitrite was added to a final concentration of 5 mM to the reduced *Ng*CuNiR and spectra were acquired during a 30 min incubation period. The reoxidation of *Ng*CuNiR after sodium nitrite addition was assessed at pH 5.5 in 100 mM 2-(N-morpholino)ethanesulfonic buffer (MES), pH 6.0 and pH 7.3 in 100 mM sodium phosphate buffer, and pH 8.5 in 100 mM of N-cyclohexyl-2-aminoethanesulfonic buffer (CHES).

## 4. Conclusions

This work presents the first complete spectroscopic characterization of *Ng*CuNiR, the copper nitrite reductase from the pathogen *N. gonorrhoeae.* The recombinant *Ng*CuNiR, consisting only of the soluble domain, was successfully isolated with both copper centers occupied. The UV–visible spectrum shows the typical absorption bands at 598 nm and 460 nm, corresponding to the S(Cys) π → Cu and S(Cys) pseudo σ → Cu charge-transfer transitions, respectively, with an ε_460nm_/ε_598nm_ of 0.6, and its extinction coefficients are now reported. These absorption bands are characteristic of bluish-green CuNiRs, typically with ε_460nm_/ε_600nm_ close to or less than 1. In addition, the EPR spectrum of *Ng*CuNiR has unique features, as the T1Cu center has a rhombic signal, while the T2Cu has a small hyperfine constant. Therefore, *Ng*CuNiR is a bluish-green CuNiR with a T1Cu center of a lower symmetry and a more tetragonal structure, which may be due to its shorter Cu—S(Cys) bond and its relatively longer Cu—S(Met) bond.

The unfolding process was characterized considering a two-state model, but it is plausible to consider that it is kinetically controlled and there is no dissociation of the trimer, as observed for *A. faecalis* S-6 CuNiR [48]. In addition, the spectroscopic features of the T1Cu center of *Ng*CuNiR are maintained up to about 80 °C, as observed by CD and visible spectroscopy, while the secondary structure does not change up to 50 °C (as observed by CD in the far-UV region). This may indicate that the metal centers contribute to the high thermostability of this enzyme (characterization of the apo form of the enzyme will confirm this hypothesis). The melting temperature estimated by the different techniques is 94 °C, revealing that this is a remarkably stable protein. This feature is not unique to this CuNiR, but there are few examples of such hyperthermostable metalloproteins that are isolated from mesophilic organisms [48,66]. This feature may be related to its tertiary and quaternary structure and gives *N. gonorrhoeae* an advantage during infection.

*Ng*CuNiR is a surface-exposed protein required for *N. gonorrhoeae* viability under anaerobic conditions [33,67] and enhances bacterial survival by producing NO, which inhibits epithelial exfoliation [28]. Thus, the hyperthermostable nature of *Ng*CuNiR and higher reoxidation rates in the presence of nitrite at acidic pH provide an advantage to *N. gonorrhoeae* during infection and contribute to its proliferation in the human epithelium. Such features can be explored to identify compounds (new or reformulated) that destabilize this enzyme and reduce gonococcal survival in the human host.

## Data Availability

Additional data are presented in Supplementary Materials, and any other data are available upon request.

## Supplementary Materials

The following supporting information can be downloaded at: https://www.mdpi.com/article/10.3390/biom13081215/s1, Figure S1: Multiple primary sequence alignment of copper nitrite reductases from different microorganisms; Figure S2: Schematic representation of the construct for *Ng*CuNiR production; Figure S3: Molecular size exclusion chromatography of as-isolated *Ng*CuNiR; Figure S4: Comparison of T1Cu center geometry between different classes of CuNiRs; Figure S5: Overlay of the spectra of *Ng*CuNiR acquired at 20 °C before and after the temperature ramp; Figure S6: Thermostability of *Ng*CuNiR studied by absorption spectroscopy; Table S1: Copper–ligand distances and bond angles of T1Cu center from CuNiRs of the different classes and of pseudoazurin.
