# Supplementary material for: Biochemical Characterization of the Copper Nitrite Reductase from Neisseria gonorrhoeae
Source: Biomolecules. 2023 Aug 4;13(8):1215. doi: 10.3390/biom13081215 (PMC10452240; doi:10.3390/biom13081215)
Supplement: Supplementary file 1 [file biomolecules-13-01215-s001.zip › biomolecules-2484167-supplementary.pdf]

Supplementary Materials

Biochemical Characterization of the Copper Nitrite Reductase from *Neisseria gonorrhoeae*

Daniela S. Barreiro[1,2], Ricardo N. S. Oliveira[1,2], Sofia R. Pauleta[1,2,*]

[1] Microbial Stress Lab, UCIBIO—Applied Molecular Biosciences Unit, Department of Chemistry, NOVA School of Science and Technology, Universidade NOVA de Lisboa, 2829-516 Caparica, Portugal.

[2] Associate Laboratory i4HB—Institute for Health and Bioeconomy, NOVA School of Science and Technology, Universidade NOVA de Lisboa, 2829-516 Caparica, Portugal.

Corresponding Author:
Sofia R. Pauleta
Microbial Stress Lab, UCIBIO—Applied Molecular Biosciences Unit, Department of Chemistry, NOVA School of Science and Technology, Universidade NOVA de Lisboa, 2829-516 Caparica, Portugal.

Tel. +351 212948385, ext 10967
Fax. + 351 351 212 948 550
E-mail: sofia.pauleta@fct.unl.pt

INDEX

S1. Primary sequence alignment S2
S2. Schematic representation of the construct used to heterologously produce *Ng*CuNiR S3
S3. Oligomeric state of *Ng*CuNiR S3
S4. Geometry of T1Cu center of NgCuNiR S4
S5. Thermostability of *Ng*CuNiR S5

S1. Primary sequence alignment

```
                               10        20        30        40        50        60
                                |         |         |         |         |         |
NgAniA      MKRQALAAMIASLFALAACGGEQAAQAPAETPAASAEAASSAAQATAETPAGELPVIDAV
AxCuNiR     -------------------MNALRPTLLAAALAFTMAAGTAWAQDADKLPHTKVTLVAPP
AfCuNiR     -MAEQMQISRRTILAGAALAGALAPVLATTSAWGQGAVRKATAAEIAALPRQKVELVDPP
RsCuNiR     -----MFTRRAALVGAAALASAPLVIRTAGAQEAPAQLASAAPVDLSNLPRVKHTLVPPP
GbCuNiR     ------MNRNVYAVLSTVLAASLLAACNSGGEQVKAESKNKTAATQQSEPNVIAAHKGVN
                                        :          .  .      *

                   70        80        90       100       110
                    |         |         |          |         |
NgAniA      TTHAPEVPPAIDRDYPAKVRVKMETVEKTMKMDDG-VEYRYWTFDGDVPGRMIRVREGDT
AxCuNiR     QVHPHEQATKSG---PKVVEFTMTIEEKKMVIDDKGTTLQAMTFNGSMPGPTLVVHEGDY
AfCuNiR     FVHAHSQVAEGG---PKVVEFTMVIEEKKIVIDDAGTEVHAMAFNGTVPGPLMVVHQDDY
RsCuNiR     FAHAHEQVAASG---PVINEFEMRIIEKEVQLDED-AYLQAMTFDGSIPGPLMIVHEGDY
GbCuNiR     QAPVPLKMERVG---PHDVHIEMTAQITDIEIDKG-KIYKAWTFNGQAPGPLVVVNEGDT
             .         .    *    .. *     . : :*.      :   :*:*   **   : *.:.:*

           120       130       140       150       160       170
            |         |         |         |         |         |
NgAniA      VEVEFSNNPSSTVPHNVDFHAATGQGGGAAATFTAPGRTSTFSFKALQPGLYIYHCAVAP
AxCuNiR     VQLTLVNPATNAMPHNVDFHGATGALGGAKLTNVNPGEQATLRFKADRSGTFVYHCAPEG
AfCuNiR     LELTLINPETNTLMHNIDFHAATGALGGGGLTEINPGEKTILRFKATKPGVFVYHCAPPG
RsCuNiR     VELTLINPPENTMPHNIDFHAATGALGGGGLTLINPGEKVVLRFKATRAGAFVYHCAPGG
GbCuNiR     IHFTLKN-MDPVVPHSMDFHAVHAS-PSKDFIDVMPNKSGTFTYPANKPGVFMYHCGTKP
            :.. : *     .: *.:***.. .    .        *..    : : * :.* ::***.

             180       190       200              210       220
              |         |         |                |         |
NgAniA      --VGMHIANGMYGLILVEPKEGLP-------KVDKEFYIVQGDFYTKGKKG---------
AxCuNiR     -MVPWHVVSGMSGTLMVLPRDGLKDPQGKPLHYDRAYTIGEFDLYIPKGPDGKYKDYATL
AfCuNiR     -MVPWHVVSGMNGAIMVLPREGLHDGKGKALTYDKIYYVGEQDFYVPRDENGKYKKYEAP
RsCuNiR     PMIPWHVVSGMAGCIMVLPRDGLKDHEGKPVRYDTVYYIGESDHYIPKDEDGTYMRFSTP
GbCuNiR     --VLQHIANGMHGVIIVKPKNGYP----TDKEVDREYVLIQNEWYKYNDMN-------DF
              :  *:..** * ::* *::*             *  : : : : *      .

                   230       240       250       260       270       280
                    |         |         |         |         |         |
NgAniA      AQGLQPFDMDKAVAEQPEYVVFNGHVGSIAGDNALKAKAGETVRMYVGNGGPNLVSSFHV
AxCuNiR     AESYG-DTVQVMRTLTPSHIVFNGKVGALTGANALTAKVGETVLLIHSQ--ANRDTRPHL
AfCuNiR     GDAYEDTVVKVMRTLTPTHVVFNGAVGALTGDKAMTAAVGEKVLIVHSQ--ANRDTRPHL
RsCuNiR     SEGYE-DMVAVMDTLIPSHIVFNGAVGALTGEGALKAKVGDNVLFVHSQ--PKRDSRPHL
GbCuNiR     QNGVPSYVVFSSKALKPGDPNTNGDTFTLK-EKPLLAKVGEKIRLYINNVGPNEVSSFHV
             :.      :      :   *       ** . ::      .: * .*:.: :  .:  .:  :   *:

                   290          300       310       320       330
                    |            |         |         |         |
NgAniA      IGEIFDKVYVEGGKLIN--ENVQSTIVPAGGSAIVEFKVDIPGSYTLVDHSIFRAFNKGA
AxCuNiR     IGGHGDWVWETGKFANPPQRDLETWFIRGGSAGAALYTFKQPGVYAYLNHNLIEAFELGA
AfCuNiR     IGGHGDYVWATGKFNTPPDVDQETWFIPGGAAGAAFYTFQQPGIYAYVNHNLIEAFELGA
RsCuNiR     IGGHGDLVWETGKFHNAPERDLETWFIRGGTAGAALYKFLQPGVYAYVNHNLIEAVHKGA
GbCuNiR     VGTVFDDVYLDGN-PNNHLQGMQTVMLPASGGAVVEFTVTRPGTYPIVTH-QFNHAQKGA
            :*    *  *:   *            . :: :: .. .. . :..    ** *.  :  *::::.  . **

           340       350       360       380       490       400
            |         |         |         |         |         |
NgAniA      LGQLKVEGAENPEIMTQKLSDTAYAGSGAASAPAASAPAASAPAASASEKSVY
AxCuNiR     AGHIKVEGKWNDDLMKQIKAPAPIPR---------------------------
AfCuNiR     AAHFKVTGEWNDDLMTSVLAPSGT-----------------------------
RsCuNiR     TASVLVEGEWDNDLMEQVWPPVGLTG---------------------------
GbCuNiR     VAMLKVTETGEDDGTETSGH---------------------------------
             .  . *     : :
```

Figure S1. Multiple primary sequence alignment of copper nitrite reductases from *N.* gonorrhoeae (*Ng*AniA, WP_003700168.1), *A. xylosoxidans* (*Ax*CuNiR, BAA33678.1), *A. faecalis* (*Af*CuNiR, D13155.1), *R. sphaeroides* (*Rs*CuNiR, AAB05767.1) and *G. thermodenitrificans* (*Gb*CuNiR, ATO36858.1). Homology code: asterisks, colons or stops below the sequence indicate identity, high conservation, or conservation of the amino acids, respectively. Legend: underlined – signal peptide, bold - palmitoylation site, grey - peptidase II cleavage site, black - tandem repeated regions, orange - residues coordinating T2Cu center, blue - residues coordinating T1Cu center, pink - catalytically important residues, Aps99 and His240. Sequence alignment performed with ClustalOmega (EMBL-EBI).

S2. Schematic representation of the construct used to heterologously produce *Ng*CuNiR

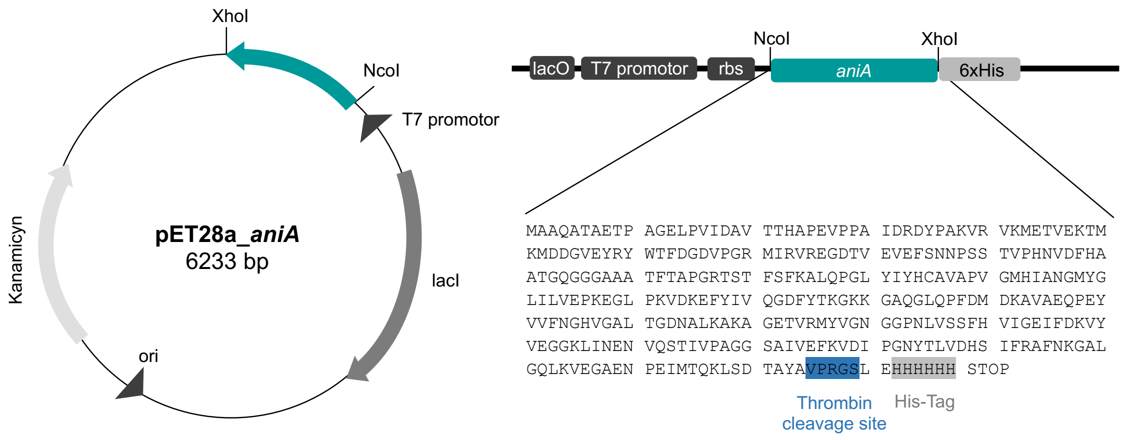

Figure S2. Schematic representation of the construct for *Ng*CuNiR production.

S3. Oligomeric state of *Ng*CuNiR

The oligomeric state of *Ng*CuNiR was confirmed by size exclusion chromatography to be a homotrimer. In fact, the apparent molecular mass of *Ng*CuNiR was determined to be 96±10 kDa, and since the expected molecular mass of the trimer is 109.5 kDa (3 x 36384 Da from the polypeptide chain plus 6 x 63.55 Da from the copper atoms) and of the monomer is 36.5 kDa, the oligomerization state would be 96/36.5 = 2.6. The low value can be explained knowing that the molecular mass determined by gel filtration if affected by the shape and hydrodynamic radius of the protein.

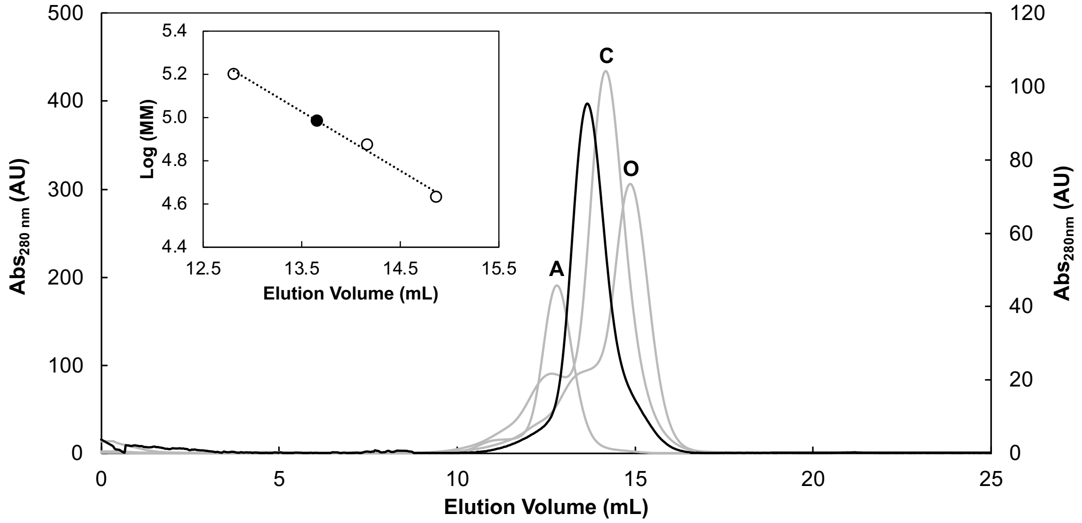

Figure S3. Molecular size exclusion chromatography of as-isolated *Ng*CuNiR (96 kDa, black line). In grey are the elution profiles of standards proteins used to estimate the apparent molecular weight: Aldolase (**A**, 158 kDa), Conalbumin (**C**, 75 kDa), Ovalbumin (**O**, 44 kDa). Inset: Logarithm of the molecular weight of the standard proteins (open circles) as a function of elution volume used to determine the molecular weight of *Ng*CuNiR (filled circle). Experimental conditions are described in Materials and Methods.

S4. Geometry of T1Cu center of *Ng*CuNiR

The geometry of T1Cu centers of blue *Alcaligenes xylosoxidans* CuNiR, bluish-green *Ng*CuNiR and green *Achromobacter cycloclastes* CuNiR are compared in Figure S4 and Table S1. In Table S1 is also presented the bond length and angles of the copper center of *Paracoccus pantotrophus* pseudoazurin.

Table S1. Copper-ligand distances and bond angles of T1Cu center from CuNiRs of the different classes and of pseudoazurin.

**CuNiR class**	**Blue**	**Bluish-Green**	**Green**	**Pseudoazurin**
PDB identifier	1OE1 (1.04 Å)	1KBW (2.4 Å)	2BW4 (0.9 Å)	3ERX (1.4 Å)
Distance (Å)				
His_1-Cu	2.02	1.99	2.04	2.13
His_2-Cu	2.20	2.10	2.23	2.11
Cys-Cu	2.03	1.90	2.03	2.13
Met-Cu	2.45	2.61	2.49	2.75
Angle (°)				
His_1-Cu-Cys	122	128	127	136
His_1-Cu-His_2	101	108	101	106
His_1-Cu-Met	88	82	86	82
Cys-Cu-His_2	114	105	108	113
Cys-Cu-Met	114	108	108	111
His_2-Cu-Met	116	128	127	108
Dihedral angle (φ)[a]	74.3	61.6	65.5	76.2

[a] Dihedral angle (φ) measured between the planes $His_1N^{\delta1}$-Cu-$His_2N^{\delta1}$ and $MetS^{\delta}$-Cu-$CysS^{\gamma}$.

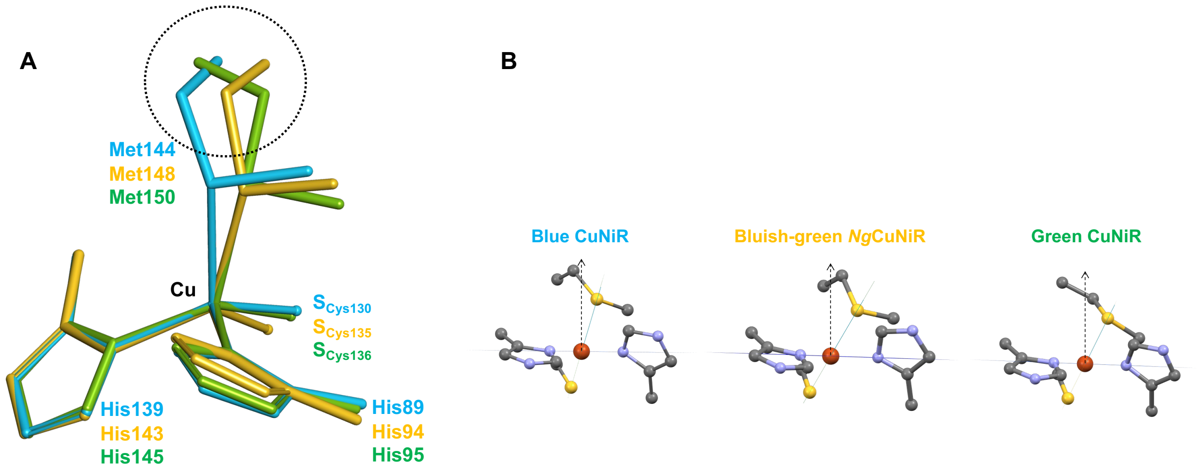

Figure S4. Comparison of T1Cu center geometry between different classes of CuNiRs. In Panel (A) is shown the superposition of the structures of the blue CuNiR from *Alcaligenes xylosoxidans* (PDB ID 1OE1) (in blue), the bluish-green CuNiR from *Neisseria gonorrhoeae* (PDB ID 1KBW) (in orange), and of the green CuNiR from *Achromobacter cycloclastes* (PDB ID 2BW4) (in green). The circle highlights the different conformation of the Met ligand which adopts a "*gauche*" conformation in blue and bluish-green CuNiRs and a "*trans* (*anti*)" conformation in green CuNiR. In Panel (B) is shown the deviation of axial ligand Met from the His_1-Cu-His_2 plane, using the same structures.

S5. Thermostability of *Ng*CuNiR

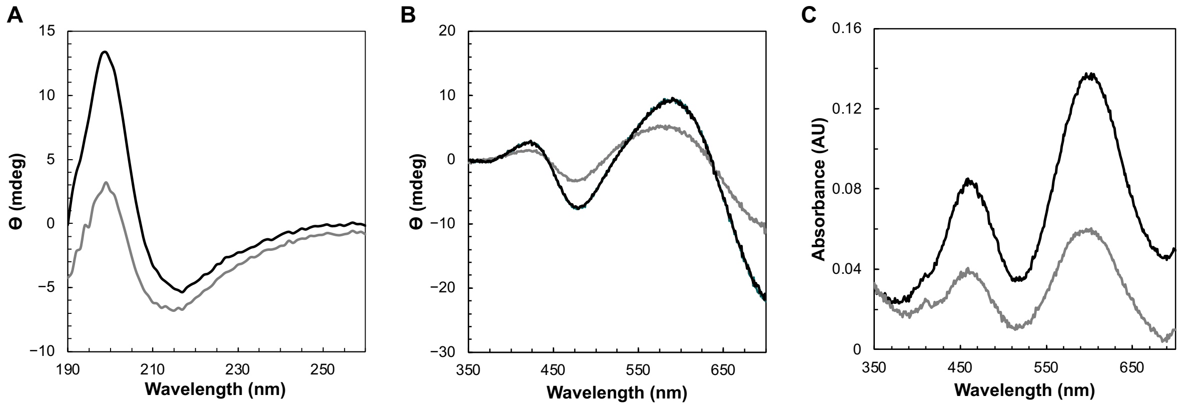

Figure S5. Overlay of the spectra of *Ng*CuNiR acquired at 20 °C before and after the temperature ramp. (**A**) CD spectra in the far-UV region; (**B**) CD spectra in the visible region; (**C**) Absorption spectra in the visible region.

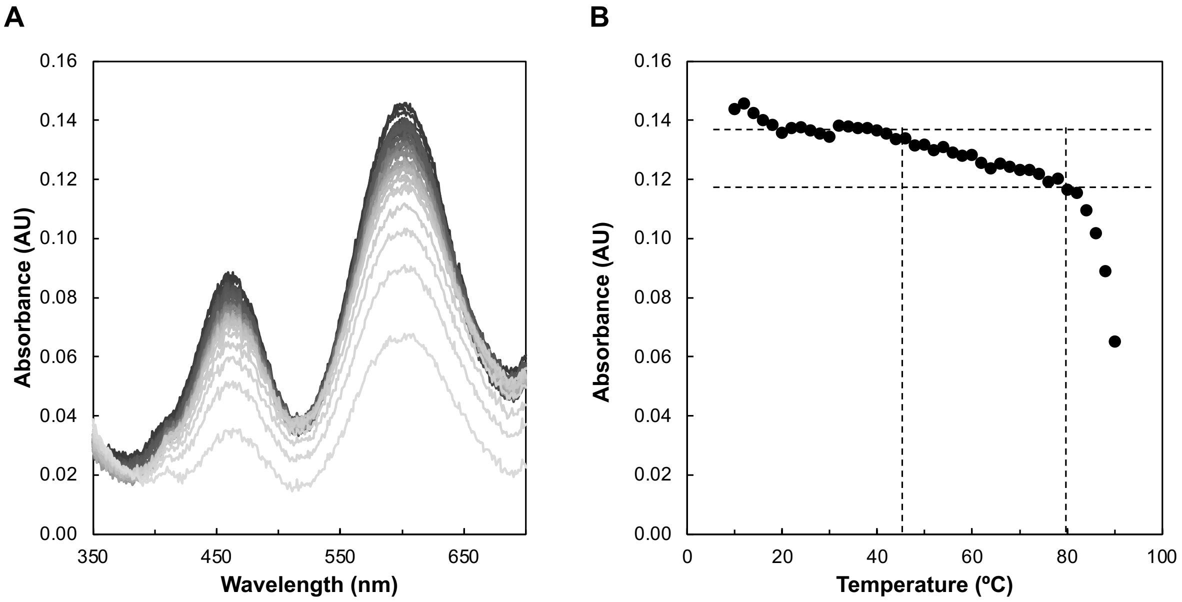

Figure S6. Thermostability of *Ng*CuNiR studied by absorption spectroscopy. (**A**) UV-visible spectra of as-isolated *Ng*CuNiR as a function of temperature, from black (10 °C) to light grey (90 °C). (**B**) Profile of the absorbance at 598 nm as a function of temperature. These spectra were collected simultaneously with the CD spectra in the visible region presented in Figure 4B.
